# Genetic variation in the aquaporin TONOPLAST INTRINSIC PROTEIN 4;3 modulates maize cold tolerance

**DOI:** 10.1111/pbi.14426

**Published:** 2024-07-18

**Authors:** Rong Zeng, Xiaoyan Zhang, Guangshu Song, Qingxue Lv, Minze Li, Diyi Fu, Zhuo Zhang, Lei Gao, Shuaisong Zhang, Xiaohong Yang, Feng Tian, Shuhua Yang, Yiting Shi

**Affiliations:** ^1^ State Key Laboratory of Plant Environmental Resilience, College of Biological Sciences, Frontiers Science Center for Molecular Design Breeding, Center for Crop Functional Genomics and Molecular Breeding China Agricultural University Beijing China; ^2^ Jilin Academy of Agricultural Sciences (Northeast Agricultural Research Center of China) Changchun China; ^3^ National Maize Improvement Center, Frontiers Science Center for Molecular Design Breeding, Department of Plant Genetics and Breeding China Agricultural University Beijing China

**Keywords:** cold stress, maize, *TIP4;3*, natural variation, stomatal movement, reactive oxygen species

## Abstract

Cold stress is a major abiotic stress that threatens maize (*Zea mays* L.) production worldwide. Understanding the molecular mechanisms underlying cold tolerance is crucial for breeding resilient maize varieties. Tonoplast intrinsic proteins (TIPs) are a subfamily of aquaporins in plants. Here, we report that TIP family proteins are involved in maize cold tolerance. The expression of most *TIP* genes was responsive to cold stress. Overexpressing *TIP2;1*, *TIP3;2* or *TIP4;3* reduced the cold tolerance of maize seedlings, while loss‐of‐function mutants of *TIP4;3* exhibited enhanced cold tolerance. Candidate gene‐based association analysis revealed that a 328‐bp transposon insertion in the promoter region of *TIP4;3* was strongly associated with maize cold tolerance. This transposon insertion conferred cold tolerance by repressing *TIP4;3* expression through increased methylation of its promoter region. Moreover, TIP4;3 was found to suppress stomatal closure and facilitate reactive oxygen species (ROS) accumulation under cold stress, thereby inhibiting the expression of cold‐responsive genes, including *DEHYDRATION‐RESPONSIVE ELEMENT BINDING FACTOR 1* (*DREB1*) genes and a subset of peroxidase genes, ultimately attenuating maize cold tolerance. This study thus elucidates the mechanism underlying TIP‐mediated cold tolerance and identifies a favourable *TIP4;3* allele as a potential genetic resource for breeding cold‐tolerant maize varieties.

## Introduction

Maize (*Zea mays* L.) is one of the most widely cultivated crops worldwide. Maize originated in the tropics and is especially sensitive to cold stress (yyGreaves, 1996). Cold stress negatively affects seed germination, seedling development and growth, culminating in lower grain yields (Verheul *et al*., 1996). Especially in regions with high‐latitude and altitude, cold stress during early spring presents a significant meteorological threat to maize production (Farooq *et al*., 2009). Therefore, enhancing the cold tolerance of maize during the germination and seedling stages can help mitigate the negative effects of temperature fluctuations.

The cold tolerance of maize is a complex trait governed by multiple quantitative trait loci (QTLs) throughout its genome. Various studies employing QTL mapping and genome‐wide association study (GWAS) methods have identified several genomic regions containing single nucleotide polymorphisms (SNPs) and insertions/deletions (InDels) associated with maize cold tolerance (Leipner *et al*., 2008; Zhang *et al*., 2020; Zhou *et al*., 2022). Metabolite GWAS has also played a significant role in elucidating the genetic basis of metabolic diversity during maize cold stress response (Pranneshraj *et al*., 2022; Zhu *et al*., 2023). For example, natural genetic variations in genes such as *INDUCER OF CBF EXPRESSION 1* (*ICE1*), which encodes a basic helix–loop–helix (bHLH) transcription factor, have been associated with variations in nitrogen metabolism and cold tolerance in maize (Jiang *et al*., 2022). Recent lipidomic analyses have implicated a subset of key enzymes involved in lipid metabolism in maize cold tolerance, expanding the understanding of the metabolic mechanisms underlying cold response in maize (Gao *et al*., 2023). Additionally, advancements in transgenic technology have facilitated the identification of genes regulating maize cold tolerance through reverse genetic approaches. A subset of crucial genes involved in maize cold tolerance have been identified, including DEHYDRATION‐RESPONSIVE ELEMENT BINDING FACTOR1 (DREB1), also reported as C‐REPEAT BINDING FACTOR (CBF), type‐A response regulator 1 (ZmRR1), cellulose synthetase (CesA), mitogen‐activated protein kinases (MPK2 and MPK8) and basic leucine zipper 68 (bZIP68) (Li *et al*., 2022; Yang *et al*., 2023; Zeng *et al*., 2021).

The cold signalling pathway mediated by CBF/DREB1 key transcription factors have been extensively studied across plant species (Liu *et al*., 2018; Shi *et al*., 2018). In *Arabidopsis thaliana*, three CBF/DREB1 genes play a crucial role in regulating the expression of cold‐regulated (*COR*) genes. In maize, overexpression of *DREB1* genes, such as *DREB1A*, *DREB1.5*, *DREB1.7* and *DREB1.10*, enhances cold tolerance (Han *et al*., 2020; Li *et al*., 2022; Zeng *et al*., 2021), suggesting the conserved role of DREB1s in cold tolerance in different plant species (Yang *et al*., 2023). Natural variations in the *ZmRR1* and *bZIP68* were shown to be associated with cold tolerance through DREB1 pathway. For instance, a natural variant of ZmRR1 with a deletion containing 15‐amino acid residues in the coding region enhances cold tolerance by stabilizing ZmRR1 and promoting the expression of *COR* genes, e.g., *DREB1* and *CesA* genes (Zeng *et al*., 2021). Conversely, bZIP68 acts as a negative regulator of maize cold tolerance by directly suppressing the expression of *DREB1* genes. MPK8 phosphorylates bZIP68 to enhance its protein stability and DNA‐binding affinity (Li *et al*., 2022). Intriguingly, *bZIP68* underwent selection during early domestication and the superior allele in teosinte was employed in maize (Li *et al*., 2022), suggesting that new gene resource for cold tolerance trait could be mined from the teosinte. Although numerous studies employing various strategies have identified potential candidate genes, validation of these candidates remains limited, thus constraining the comprehension of the molecular and genetic basis of maize cold tolerance.

Aquaporins (AQPs) belong to the main intrinsic protein family and regulate the water balance of plant cells and tissues by facilitating water transport across membranes (Johansson *et al*., 2000). Plant AQPs can transport small molecules such as CO_2_, hydrogen peroxide (H_2_O_2_) urea, ammonia, glycerol, silicon, boron, and arsenic (Sun *et al*., 2024). Emerging studies has shown that AQPs play a crucial role in mitigating damage caused by abiotic stresses, including salinity, alkali stress, drought and hypoxia stress, in many plant species (Afzal *et al*., 2016; Sudhakaran *et al*., 2021). For example, in Arabidopsis, NOD26‐LIKE INTRINSIC PROTEIN 2;1 (NIP2;1) acts as a lactic acid efflux channel to enhance plant survival during the hypoxia stress (Beamer *et al*., 2021). In rice (*Oryza sativa*), overexpressing *RICE WATER CHANNEL 3* (*RWC3*), which encodes a plasma membrane intrinsic protein 1 (PIP1) type AQP, increased root hydraulic conductivity (Lpr) and improved water status under drought stress (Lian *et al*., 2004). In rose (*Rosa* sp.), drought‐induced phosphorylation of PIP2;1 promotes the nuclear translocation of the membrane‐tethered MYB transcription factor PHD TYPE TRANSCRIPTION FACTOR WITH TRANSMEMBRANE DOMAINS (PTM), thereby enhancing drought tolerance (Zhang *et al*., 2019). Similarly, tobacco (*Nicotiana tabacum*) AQP1 enhances water use efficiency, hydraulic conductivity and yield under salt stress (Sade *et al*., 2010). Conversely, an *Ospip2;1* mutant was sensitive to alkali stress, with a reduced survival rate and poor growth (Zhang *et al*., 2023). Nevertheless, whether and how AQPs involve in cold tolerance in plants remains poorly understood.

In this study, we uncovered the negative role of tonoplast intrinsic protein (TIP) family proteins in maize cold tolerance by influencing stomatal movement and reactive oxygen species (ROS) accumulation. We identified a 328‐bp transposon insertion in the promoter region of *TIP4;3*. This insertion increased methylation levels, resulting in the repression of *TIP4;3* transcription and consequently enhanced cold tolerance in maize. These findings highlight a crucial genetic target for development of cold‐tolerant maize varieties.

## Results

### Three TIPs have a negative effect on the cold tolerance of maize

**Figure 1:**
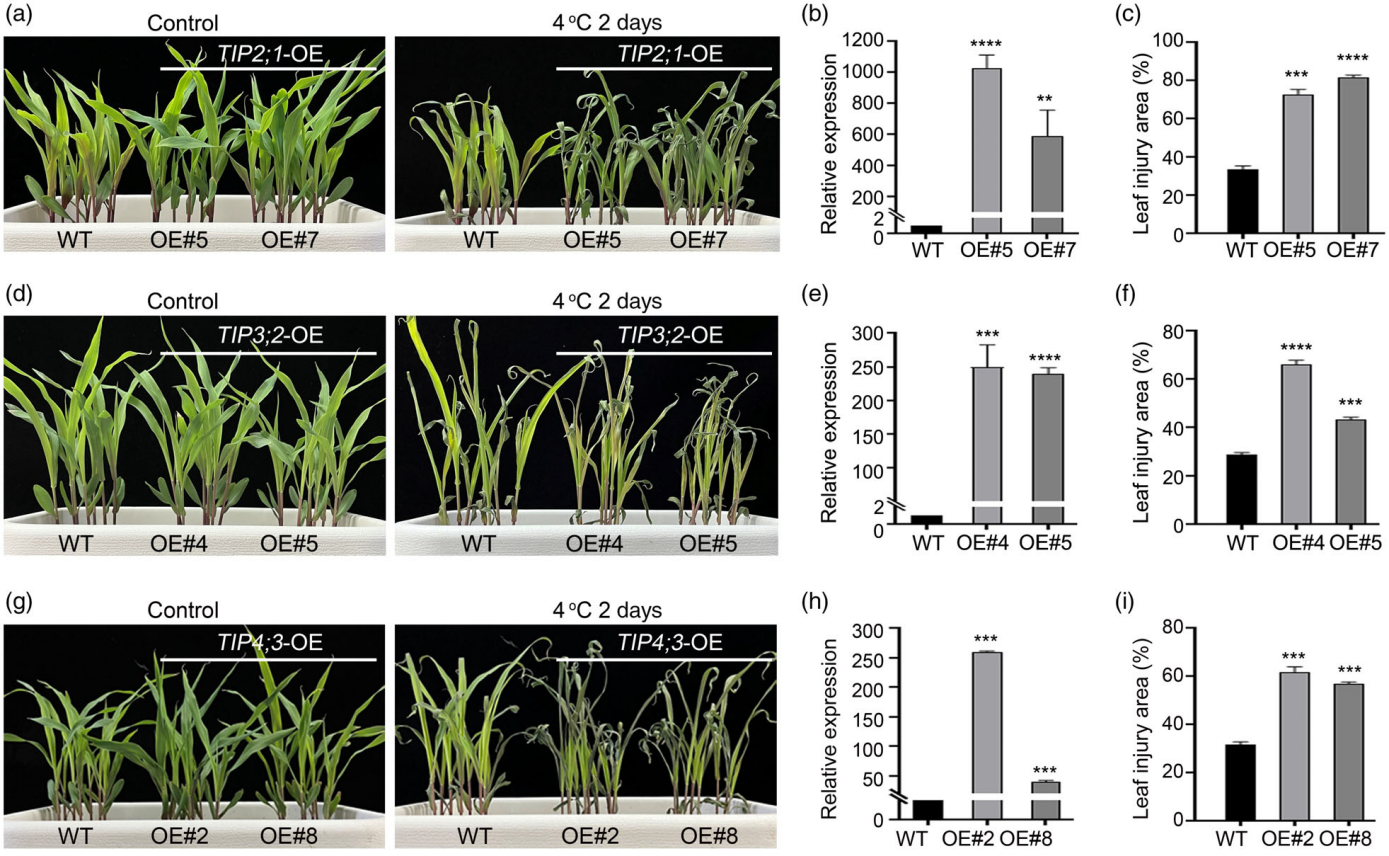
Analysis of cold tolerance in *TIP2;1*, *TIP3;2* and *TIP4;3*‐overexpressing transgenic plants. (a–c) Cold‐tolerance phenotypes (a), relative *TIP2;1* expression levels (b), and leaf injury area (c) of wild‐type (WT) and *TIP2;1*‐overexpressing transgenic plants (OE#5, OE#7) after cold treatment. (d–f) Cold‐tolerance phenotypes (d), relative *TIP3;2* expression levels (e) and leaf injury area (f) of WT and *TIP3;2*‐overexpressing transgenic plants (OE#4, OE#5) after cold treatment. (g–i) Cold‐tolerance phenotypes (g), relative *TIP4;3* expression levels (h), and leaf injury area (i) of WT and *TIP4;3*‐overexpressing transgenic plants (OE#2, OE#*8*) after cold treatment. In (a, d, g), 14‐day‐old seedlings grown at 25 °C were incubated at 4 °C for 2 days. Representative photographs were taken after 2 days of recovery at 25 °C. In (b, c, e, f, h, i), Error bars represent mean ± SD (standard deviation). *P < 0.05, **P < 0.01, ***P < 0.001, ****P < 0.0001 (two‐sided *t*‐test).

To identify the components involved in cold tolerance in maize, we previously conducted a screening on transgenic maize plants overexpressing more than 700 maize genes in the background of the LH244 inbred line under cold treatment (Zeng *et al*., 2021). Among these genes, we found that three *TIP* genes encoding tonoplast aquaporin proteins showed a negative effect on the cold tolerance of maize seedlings (Chaumont *et al*., 2001) (Figure 1a–i). Specifically, maize seedlings overexpressing *TIP2;1*, *TIP3;2*, or *TIP4;3* under the control of *Ubi* promoter exhibited impaired cold tolerance, with a higher area of leaf injury compared to wild‐type LH244 following cold treatment (Figure 1a–i). These findings suggest a negative role for these *TIP* genes in maize cold tolerance.

**Figure 2:**
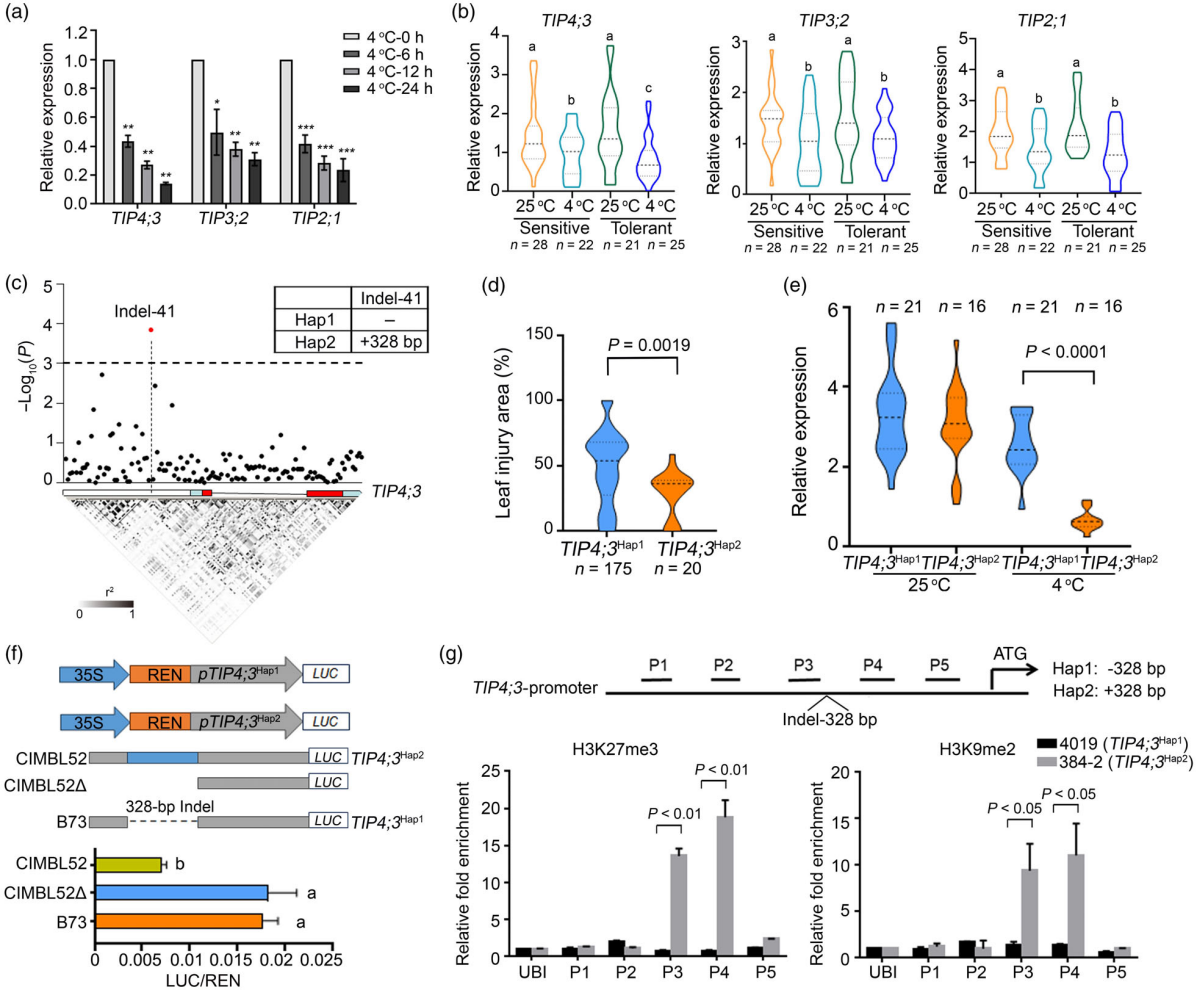
A 328‐bp insertion (InDel‐41) in *TIP4;3* is significantly associated with maize chilling tolerance. (a) Relative expression levels of *TIP2;1*, *TIP3;2* and *TIP4;3* after cold treatment at 4 °C for 0, 6, 12 and 24 h. Samples from seedlings maintained at permissive conditions (25 °C) were collected as a 0‐h time point. (b) The gene expression of *TIP4;3*, *TIP3;2* and *TIP2;1* in hyper‐sensitive and hyper‐tolerant inbred lines at permissive condition (25 °C) or after cold treatment (4 °C) for 12 h. Different letters represent significant differences (*P* < 0.05, one‐way ANOVA). “*n*” represents the number of inbred lines. (c) Local Manhattan plot showing the association analysis of genetic variation at the *TIP4;3* locus with chilling tolerance in maize and the pattern of pairwise linkage disequilibrium of DNA polymorphisms. A diagram of the *TIP4;3* locus is shown. The white, light blue and red rectangles represent 1.3‐kb promoter, UTR and coding region, respectively. The most significant Indel (Indel‐41) in the promoter is connected to its location in the locus diagram by a dotted line. The transverse dotted line represents the threshold line. (d) Area of leaf injury (%) of inbred lines harbouring the Hap1 or Hap2 haplotypes of *TIP4;3*. (e) Relative *TIP4;3* transcript levels in inbred lines harbouring the Hap1 or Hap2 haplotypes grown at 25 °C or exposed to 4 °C for 12 h. The 21 Hap1 and 16 Hap2 lines were randomly selected for gene expression analysis. (f) Dual luciferase (LUC) reporter assays, using firefly *LUC* controlled by the *TIP4;3* ^B73^, *TIP4;3* ^CIMBL52^ or *TIP4;3* ^CIMBL52Δ^ promoter in maize protoplasts under 4 °C for 3 h. (g) Chromatin immunoprecipitation followed by quantitative PCR (ChIP‐qPCR) showing the chromatin state at the *TIP4;3* ^4019^ and *TIP4;3* ^384‐2^ promoters following cold stress treatment. Anti‐H3K27me3 (left) and anti‐H3K9me2 (right) antibodies were used for immunoprecipitation. Five regions (P1–P5) of the *TIP4;3* promoter were tested; the maize *Ubiquitin* (*Ubi*) was used as a negative control.

We then performed a comprehensive analysis of the expression patterns of *TIP* genes in response to cold stress in maize. Heatmap analysis from a previous study revealed that the expression levels of nine genes out of 11 members of maize *TIP* family were detectable, and most of their transcriptions were responsive to cold stress (Alexandersson *et al*., 2005) (Figure S1a,b). Furthermore, examination of the expression of *TIP2;1*, *TIP3;2* and *TIP4;3* by RT‐qPCR showed that all these three genes were downregulated after cold treatment (Figure 2a). To explore natural variations at these three *TIP* loci, we examined their expression levels across cold‐sensitive and cold‐resistant inbred lines. Consistent with the above result, the transcript levels of these genes decreased under cold treatment (4 °C, 12 h) across all inbred lines (Figure 2b). Particularly, the cold‐mediated repression of *TIP4;3* expression was stronger on average in the cold‐tolerant inbred lines compared to the cold‐sensitive inbred lines, while the expression levels of *TIP2;1* or *TIP3;2* were comparable between the two groups of inbred lines at the same cold treatment conditions (Figure 2b). These results suggest that while *TIP2;1*, *TIP3;2* and *TIP4;3* all involved in cold stress responses, the expression level of *TIP4;3* is strongly associated with the variation in cold tolerance observed among maize inbred lines.

### Identification of a favourable *TIP4;3* allele with enhanced cold tolerance

To identify a favourable natural allele of *TIP4;3* that enhances maize cold tolerance, we re‐sequenced a 4.0‐kb genomic region containing *TIP4;3* (comprising a 1.3‐kb promoter fragment and untranslated regions [UTRs]). This analysis revealed a total of 127 SNPs (causing nonsynonymous mutations) and 13 insertions/deletions (InDels) with a minor allele frequency >5%. Subsequently, candidate gene association analysis was performed using relative leaf injury area as a phenotype reflecting the cold tolerance by TASSEL (Bonferroni threshold *P* < 1.92 × 10^−3^). Among the identified variants, a 328‐bp insertion (InDel‐41) located 489 bp upstream of the translation start site of *TIP4;3* showed the highest association with cold tolerance (*P* = 1.41 × 10^−4^) (Figure 2c). Based on this InDel, the 195 maize varieties were categorized into two haplotype groups: *TIP4;3* ^Hap1^ (175 inbred lines) and *TIP4;3* ^Hap2^ (20 inbred lines) respectively (Table S1). Notably, *TIP4;3* ^Hap1^ inbreds exhibited significantly higher leaf injury area than *TIP4;3* ^Hap2^ inbreds (*P* = 0.0019) (Figure 2d). Consistent with their cold tolerance, *TIP4;3* expression was much lower in *TIP4;3* ^Hap2^ inbreds than in *TIP4;3* ^Hap2^ inbreds under cold treatment (Figure 2e). Therefore, we designated *TIP4;3* ^Hap1^ and *TIP4;3* ^Hap2^ as the sensitive and tolerant alleles respectively.

To investigate whether the natural variation in the *TIP4;3* promoter contributes to the difference in expression levels, we performed a dual‐luciferase (LUC) reporter assay in maize protoplasts. Protoplasts were transfected with constructs containing the *LUC* reporter gene driven by the *TIP4;3* promoter derived from either the B73 (Hap1) or CIMBL52 (Hap2) inbred line. Dual‐luciferase reporter assay revealed that the *TIP4;3* ^CIMBL52^ promoter exhibited lower LUC activity, achieving only 40% of the *TIP4;3* ^B73^ promoter (Figure 2f). These findings suggest that the sequence variation in the *TIP4;3* promoter region contributes to differences in gene expression levels between the two haplotypes, thereby leading to the divergence in cold tolerance among maize inbred lines.

To investigate the effect of Indel‐41 in *TIP4;3* expression, we conducted a BLAST search against the maize transposable element (TE) database using the 328‐bp sequence of Indel‐41 as a query (http://maizetedb.org/Bmaize/). We observed that this sequence matches the *CACTA* TE family. Sequence analysis of the 328‐bp fragment revealed the presence of (5′‐CACTA‐3′) terminal inverted repeats and 11‐bp sub‐terminal repeats (TTTGCCGAGTG) (Figure S2a,b). Transposon insertions have been known to influence the methylation levels of histones, subsequently affecting gene expression (Mao *et al*., 2015). Therefore, we examined the levels of di‐ and tri‐methylation of lysine 9 or lysine 27 of histone H3 (H3K9me2 and H3K27me3) along the promoters of *TIP4;3* ^Hap1^ inbred line (4019) and *TIP4;3* ^Hap2^ inbred line (384‐2) under cold stress. We observed a much higher histone methylation level in the *TIP4;3* ^Hap2^ promoter than the *TIP4;3* ^Hap1^ promoter (Figure 2g). These results suggest that the 328‐bp insertion leads to the elevated H3K9me2 and H3K27me3 levels at the *TIP4;3* promoter region, thereby repressing *TIP4;3* transcription.

### The *TIP4;* *3* ^Hap2^ allele improves maize cold tolerance

**Figure 3:**
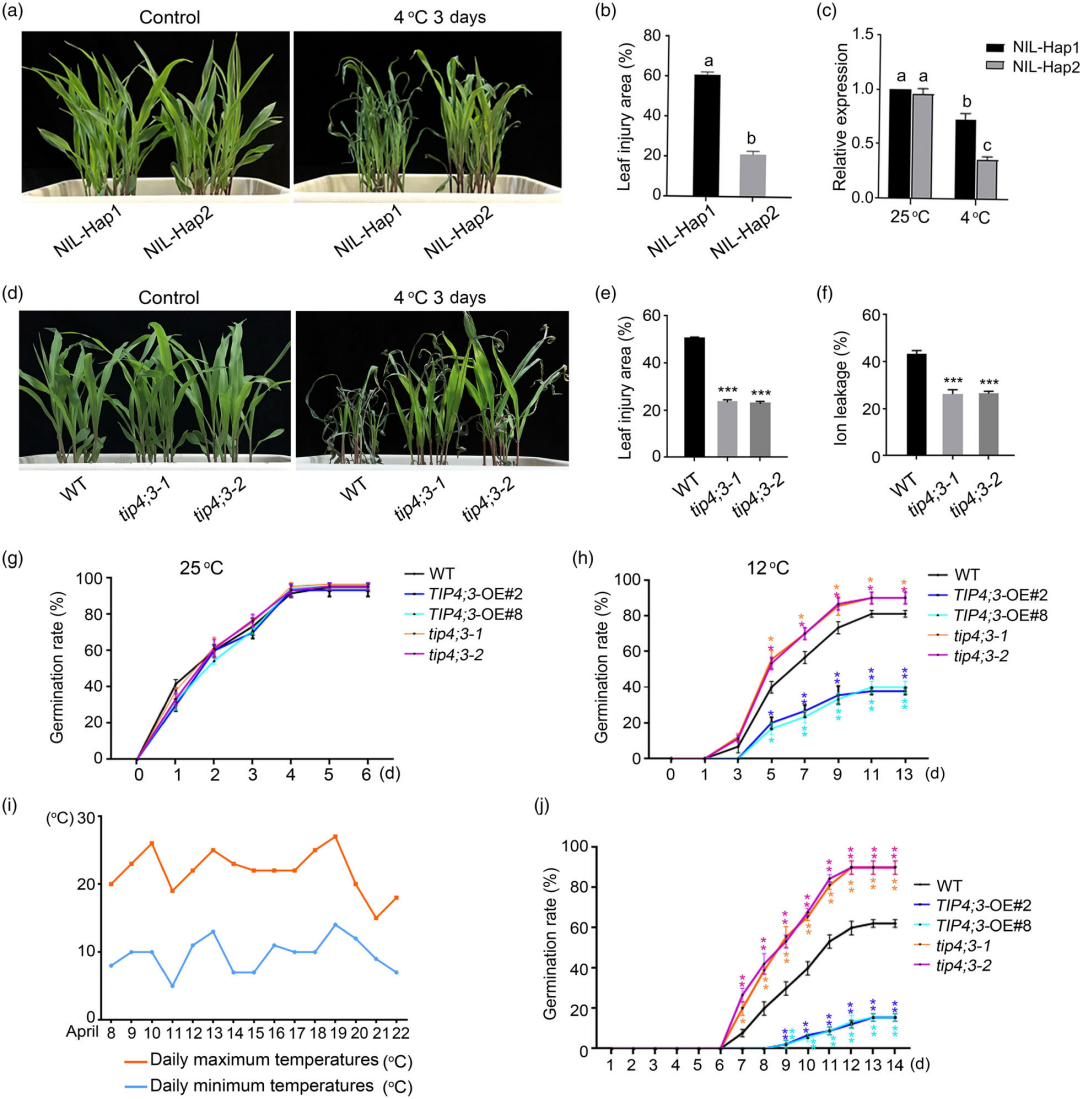
The *TIP4;3* ^Hap2^ allele improves cold tolerance in maize. (a) Chilling phenotypes of near‐isogenic lines (NILs) carrying the allele conferring cold tolerance (*TIP4;3* ^Hap2^) or the allele conferring cold sensitivity (*TIP4;3* ^Hap1^). (b) Leaf injury area of the NILs when exposed to cold stress. Bars represent 10 seedlings for each genotype in three independent experiments. Different letters represent significant differences (*P* < 0.05) determined by one‐way ANOVA with Tukey's multiple comparisons test. (c) Relative *TIP4;3* expression levels in NIL‐Hap1 (*TIP4;3* allele from 4019) and NIL‐Hap2 (*TIP4;3* allele from 384‐2). Total RNA was extracted from three seedlings in each independent experiment. Different letters represent significant differences (*P* < 0.05) determined by one‐way ANOVA with Tukey's multiple comparisons test. (d–f) Chilling phenotypes (d), leaf injury area (e), and ion leakage (f) of WT and *tip4;3* mutants. Fourteen‐day‐old seedlings grown at 25 °C were exposed to 4 °C for 3 days. Representative images were taken after 2 days of recovery at 25 °C. (g, h) Germination rates of WT, *TIP4;3*‐OE lines and *tip4;3* mutants at 25 °C (g) or 12 °C For each assay, 30 seeds of WT, *TIP4;3*‐OE lines and *tip4;3* mutants were placed in an incubator at 25 °C and 12 °C after soaking in water for 24 h. (i, j) Daily average minimum and maximum temperatures (i) and germination rates of WT, *TIP4;3*‐OE and *tip4;3* mutants in natural field conditions (j). Seeds were sown on April 8, with 30 seeds per pot; the germination rate was scored over 14 consecutive days. The daily minimum temperature was 5 °C, and the maximum temperature was 27 °C. Error bars represent mean ± SD (standard deviation) from 3 biological replicates. *P < 0.05, **P < 0.01, *** *P* < 0.001, Student's two‐sided *t*‐test.

To validate the impact of the *TIP4;3* ^Hap2^ allele on maize cold tolerance, we introgressed *TIP4;3* ^Hap2^ allele from a cold‐tolerant inbred line (384‐2) into a cold‐sensitive inbred line (4019) carrying the *TIP4;3* ^Hap1^ allele, generating near‐isogenic lines (NILs) through three generations of successive backcrossing of the F_1_ plants (384‐2 × 4019) to 4019 as the recurring parent. In each generation, *TIP4;3* ^Hap1^/*TIP4;3* ^Hap2^ heterozygous plants were selected and backcrossed to 4019. After one generation of self‐pollination, BC_3_F_2_ plants were genotyped to identify plants homozygous for the cold‐tolerant *TIP4;3* ^Hap2^ or cold‐sensitive *TIP4;3* ^Hap1^ allele (Figure 3a; Figure S3a). Evaluation of cold tolerance in these plants revealed that the NIL‐*TIP4;3* ^Hap2^ plants exhibited greater cold tolerance than NIL‐*TIP4;3* ^Hap1^ plants. Furthermore, heterozygous *TIP4;3* plants exhibited similar cold tolerance to NIL‐*TIP4;3* ^Hap2^, indicating that *TIP4;3* ^Hap2^ is a dominant allele (Figure 3a,b; Figure S3b). Moreover, lower *TIP4;3* transcript levels were detected in NIL‐*TIP4;3* ^Hap2^ plants compared to in NIL‐*TIP4;3* ^Hap1^ plants, with a more pronounced effect after cold treatment (Figure 3c). These results indicate that the two natural alleles, *TIP4;3* ^Hap1^ and *TIP4;3* ^Hap2^, result in differential expression levels of *TIP4;3*, leading to diverse cold tolerance in maize.

### *TIP4;3* compromises maize cold tolerance at the seedling and germination stages

To dissect the role of TIP4;3 in maize cold tolerance, we employed clustered regularly interspaced short palindromic repeats (CRISPR)/CRISPR‐associated nuclease 9 (Cas9)‐mediated gene editing to generate knockout mutants in the LH244 inbred line which carries the Hap1 haplotype. The *tip4;3* mutants harboured a 1‐bp deletion (*tip4;3–1*) or a 2‐bp insertion (*tip4;3–2*) in the first exon of the *TIP4;3* gene, presumed to produce premature stop codon (Figure S3c). After cold treatment, the *tip4;3* mutants displayed a reduced relative leaf injury area compared to the WT (Figure 3d–f). Additionally, we performed a cross between the *tip4;3–1* and the *tip4;3–2* mutant, and found that the F_1_ progeny displayed a cold tolerance phenotype similar to *tip4;3–1* and *tip4;3–2* (Figure S4). These results further support the notion that TIP4;3 acts as a negative regulator of cold tolerance of maize.

Cold stress usually reduces the early vigour of seeds after germination. To determine whether TIP4;3 affects the early vigour of maize seeds experiencing cold stress, we scored the germination rates of seeds at 25 °C or 12 °C in an incubator after seed hydration for 24 h using seeds from the WT, the *tip4;3* mutants, and two *TIP4;3‐*overexpression lines (*TIP4;3*‐OE#2 and #8). The germination ratio showed no clear difference among WT, *tip4;3* or *TIP4;3*‐OE plants at 25 °C (Figure 3g). When incubated at 12 °C, the germination rates of the *tip4;3* mutant seeds were higher, whereas those of the *TIP4;3*‐OE lines were notably lower than the WT (Figure 3h). We repeated this assay in natural field conditions, when the day/night temperatures were around 20 °C/10 °C: the *tip4;3* mutant seeds displayed higher germination rates than the WT, with the final rate reached 85% (*tip4;3* mutants) versus 60% (WT). In contrast, the germination rates of *TIP4;3*‐OE seeds were severely delayed compared to the WT and final germination rates remained below 20% (Figure 3i,j). Taken together, these findings suggest that TIP4;3 negatively modulates both early vigour and seedling tolerance to cold stress in maize.

### TIP4;3 acts as an aquaporin

**Figure 4:**
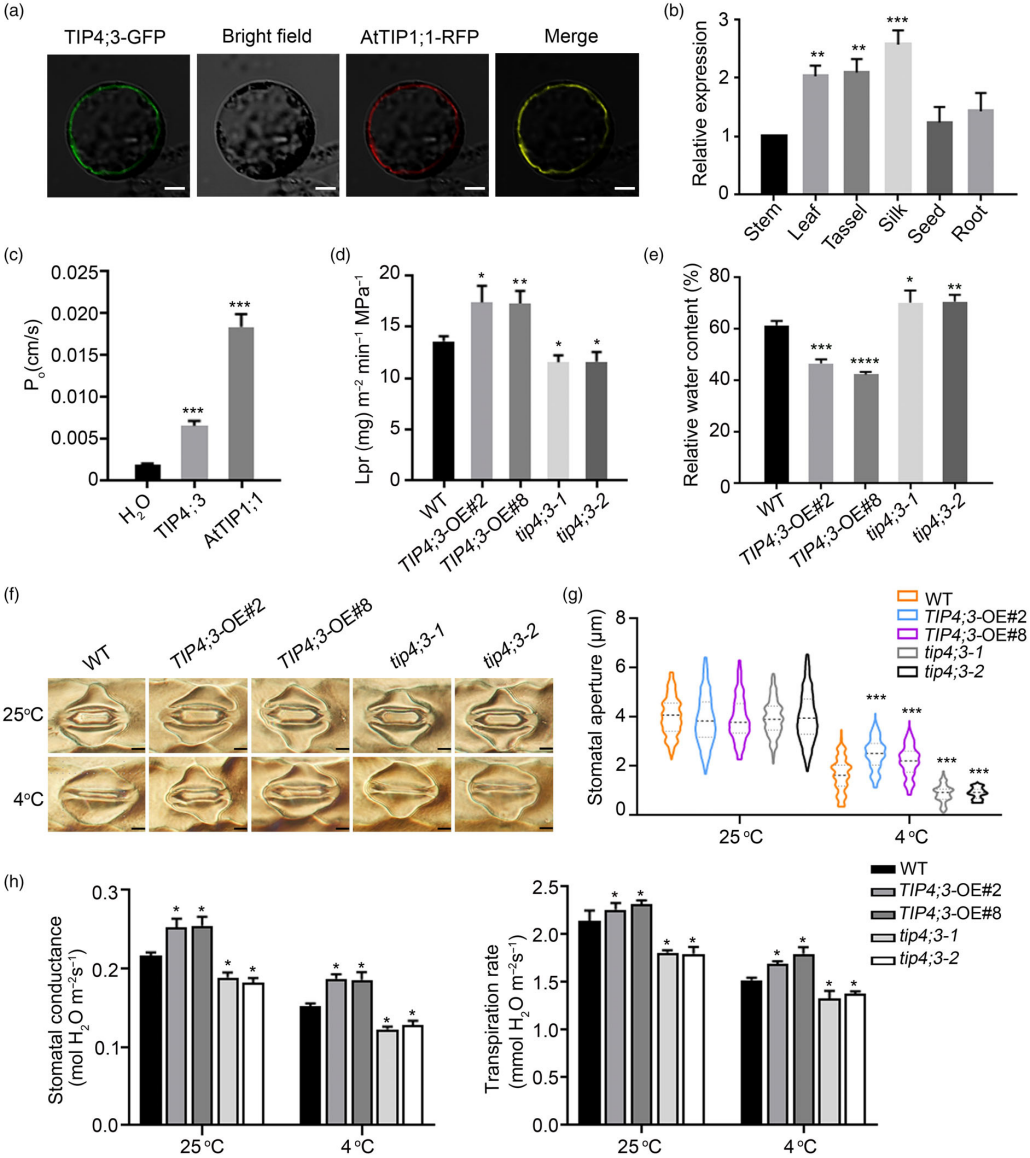
Biochemical characterization of TIP4;3 in response to cold stress. (a) Subcellular localization of TIP4;3. AtTIP1;1‐RFP (a fusion between Arabidopsis TIP1;1 and red fluorescent protein) was used as a vacuolar marker. *pSuper:TIP4;3‐GFP* and *pSuper:AtTIP1;1‐RFP* were co‐transfected into maize mesophyll protoplasts. GFP and RFP signals were visualized by confocal microscopy. Scale bars, 10 μm. (b) The relative expression of *TIP4;3* expression in different tissues of maize. (c) Osmotic water permeability (P_o_) of TIP4;3 in *Xenopus* oocytes. Water transport was assayed at 18 °C for 3 days after cRNA injection. Water transport activity analysis was performed as described in materials and methods. Oocytes injected with water were used as a negative control. AtTIP1;1, an aquaporin, was used as a positive control for water permeability. (d) Root hydraulic conductivity (Lpr) in WT, *TIP4;3*‐OE and *tip4;3* plants. Culture in hydroponic conditions was conducted for this experiment. Four‐week‐old plants were used to measure root hydraulic conductivity. (e) Relative water content of WT, *TIP4;3*‐OE and *tip4;3* plants after 4 °C treatment for 24 h. (f) Cold‐induced stomatal closure was photographed from the leaves of WT, *TIP4;3*‐OE and *tip4;3* plants before and after (4 °C). Scale bars, 5 μm. (g) Stomatal aperture in WT, *TIP4;3*‐OE and *tip4;3*. Bars represent 200 stomata in five replicates under 25 °C and 4 °C conditions. Eight‐day‐old seedlings were immersed in MES‐KOH buffer, exposed to light at 25 °C for 5 h to completely open stomata, and then treated at 4 °C for 20 min before being photographed. The dashed horizontal line represents the median, and the upper and lower dotted lines represent the third quartile and first quartile respectively. The bounds of the plot represent data density. ***P < 0.001, **P < 0.01, *P < 0.05 two‐sided *t*‐test. (h) Analysis of stomatal conductance and transpiration rate under permissive temperature and after cold treatment for 24 h. In b, c, d, e, g, h, bars represent mean ± SD (standard deviation) from three independent experiments. ****P < 0.0001, ***P < 0.001, **P < 0.01, *P < 0.05 (two‐sided *t*‐test).

*TIP4;3* encodes a vacuolar aquaporin localizing to the tonoplast in maize protoplasts, as evidenced by the overlap between the green fluorescence signal from a TIP4;3‐green fluorescent protein (GFP) fusion protein and the vacuolar membrane marker AtTIP1;1 fused to the red fluorescent protein (Figure 4a). Furthermore, TIP4;3 was found to be expressed in various maize tissues, with particularly high expression levels observed in the leaf, tassel, silk and immature cob (Figure 4b). To assess the water transport activity of TIP4;3, the *Xenopus* oocyte system was employed. Oocytes injected with the cRNA of *TIP4;3* or *AtTIP1;1* (used as a positive control) exhibited rapid swelling upon switching from an isotonic to a hypotonic buffer, displaying an increase in osmotic water permeability (Po) (Figure 4c; Figure S5). This result indicates that like AtTIP1;1, TIP4;3 possesses water transport activity.

Root hydraulic conductivity (Lpr) reflects the ability of roots to take up water (Cabrera *et al*., 2024). The Lpr of wild‐type, *TIP4;3*‐OE and *tip4;3* mutant plants was determined using the pressure‐chamber approach. Compared to the WT, *TIP4;3*‐OE plants exhibited higher Lpr (Figure 4d), indicating that overexpression of *TIP4;3* enhances root hydraulic conductivity. Conversely, *tip4;3* mutant plants displayed lower Lpr compared to the WT plants (Figure 4d), suggesting that the absence of *TIP4;3* leads to a reduction in root hydraulic conductivity. These results suggest that TIP4;3 plays a role in promoting Lpr and water uptake by roots.

### TIP4;3 regulates stomatal movement

Considering that TIP4;3 has a negative impact on cold tolerance, but plays a positive role in water uptake, we next measured the water content in WT, *TIP4;3*‐OE and *tip4;3* mutant plants in response to cold stress. We observed that the relative leaf water content was lower in *TIP4;3*‐OE lines, but higher in *tip4;3* mutants compared to the WT after cold treatment (Figure 4e). These findings prompted us to test whether TIP4;3 affects stomatal movement by measuring stomatal aperture on WT, *TIP4;3*‐OE and *tip4;3* leaves under cold treatment. Accordingly, we immersed plants in MES‐KOH buffer in the light to ensure that stomata were completely open, followed by cold treatment. While the stomatal apertures were comparable among the three genotypes under permissive (25 °C) conditions, cold stress induced different degrees of stomatal closure in WT, *TIP4;3*‐OE and *tip4;3* plants. Compared to the WT, the stomatal apertures of *TIP4;3*‐OE plants were larger, while those of the *tip4;3* mutants were smaller after cold treatment (Figure 4f,g). Furthermore, we determined the stomatal conductance and transpiration rate of 21‐day‐old WT, *TIP4;3‐OE* and *tip4;3* mutant plants grown at 25 °C and treated at 4 °C for 24 h. We observed that stomatal conductance and transpiration rate were higher in *TIP4;3‐OE* plants but lower in *tip4;3* mutants compared to the WT with or without cold treatment (Figure 4h,i). Based on these findings, we conclude that TIP4;3 attenuates cold tolerance by suppressing stomatal closure and promoting transpiration upon cold stress.

### Transcriptome profiling of *TIP4;3*

**Figure 5:**
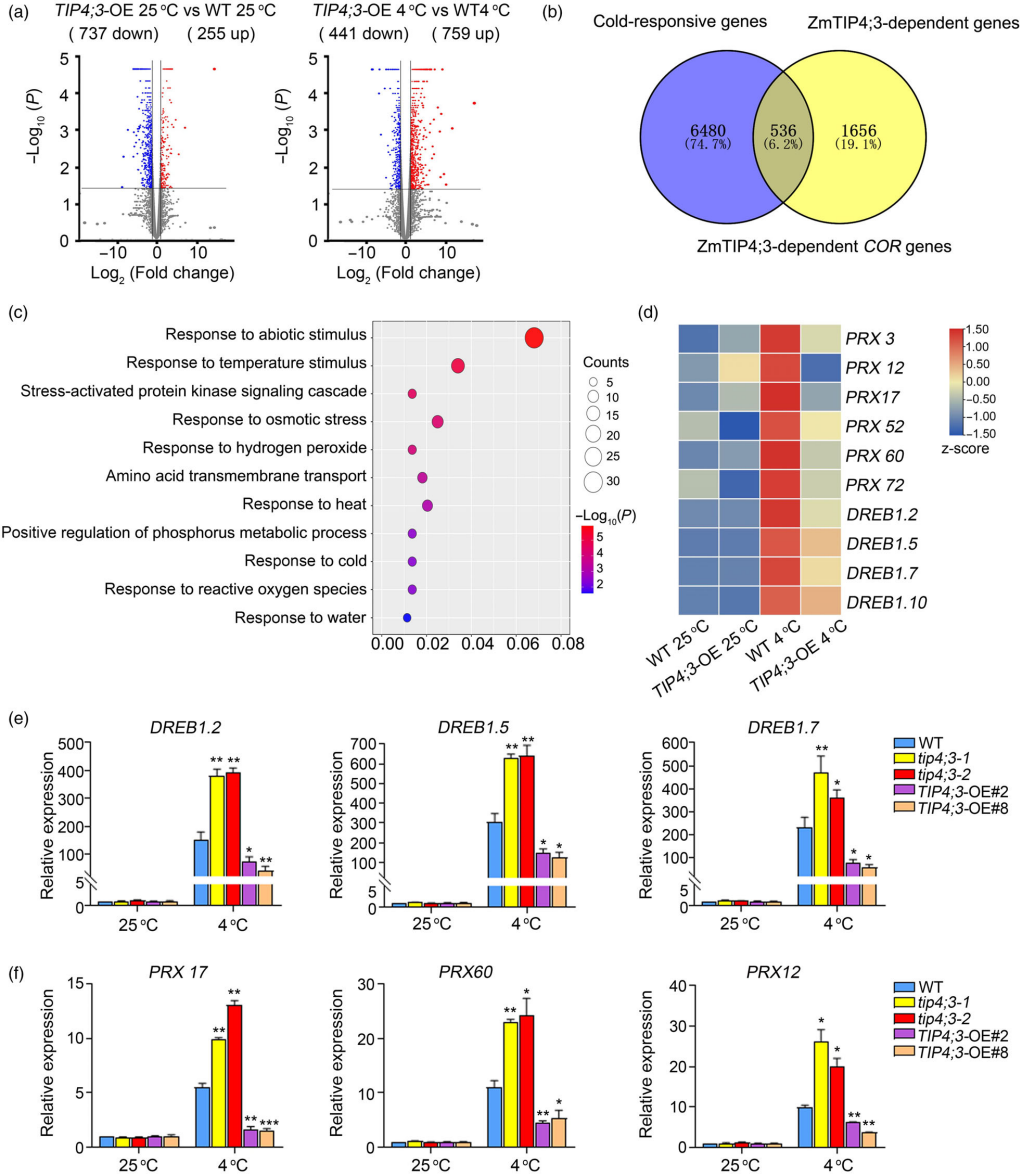
Transcriptome analysis of TIP4;3‐dependent genes. (a) Volcano plots showing the number of differentially expressed genes (DEGs) between WT and *TIP4;3*‐OE plants under control conditions (25 °C) or following cold treatment (25 °C). DEGs were identified using a *P*‐value <0.05 and absolute log_2_ (fold‐change) value ≥1 as criteria. (b) Venn diagram showing the extent of overlap between TIP4;3‐dependent genes and *COLD‐REGULATED* (*COR*) genes. (c) Gene ontology term enrichment analysis of TIP4;3‐dependent *COR* genes. (d) Heatmap representation of expression levels for some DEGs modulated by TIP4;3. FPKM values were Z‐score normalized. (e, f) Relative expression levels of *DREB1.2*, *DREB1.5*, *DREB1.7*, *PRX12*, *PRX17* and *PRX60* in WT, *TIP4;3*‐OE lines and *tip4;3* mutants under cold stress. Error bars represent mean ± SD (standard deviation) (*n* = 3; two‐sided *t*‐test). **P < 0.01, *P < 0.05.

To further investigate the role of TIP4;3 in cold tolerance of maize, we conducted transcriptome deep sequencing (RNA‐seq) using 2‐week‐old seedlings of WT and *TIP4;3*‐OE plants grown at 25 °C treated or not at 4 °C for 12 h in three independent experiments. We identified differentially expressed genes (DEGs) based on the criteria of a significant difference (*P* < 0.05) with an absolute fold‐change ≥2, yielding 2620 DEGs in the WT seedlings after exposure to cold (4 °C) compared to the WT seedlings without cold treatment (25 °C) (Table S2). We defined TIP4;3‐affected genes as those DEGs between *TIP4;3*‐OE and WT plants based on the criteria fold change ≥2 and *P* < 0.05 under permissive conditions (25 °C) or cold treatment (4 °C). We identified a total of 2192 genes affected by TIP4;3, 992 genes (255 up and 737 down) at 25 °C (Figure 5a; Table S3) and 1200 (759 up and 441 down) after cold treatment (Figure 5a; Table S4). Based on the defined cold‐responsive transcriptome (Zhang *et al*., 2017), 536 out of these 2192 genes showed significant changes in response to cold treatment; we refer to these genes as *COR* genes affected by TIP4;3 (Figure 5b).

Gene ontology (GO) term analysis indicated that the genes affected by TIP4;3 are involved in various biological processes, including response to temperature stimuli, osmotic stress, hydrogen peroxide and ROS (Figure 5c). We noticed that the expression of several *DREB1*s, key genes promoting maize cold tolerance, was affected by TIP4;3 (Figure 5d). Indeed, the expression of *DREB1.2*, *DREB1.5* and *DREB1.7* was remarkably lower in the *TIP4;3*‐OE lines, but higher in the *tip4;3* mutants compared to the WT (Figure 5e). Notably, we also observed that a subset of genes encoding peroxidases responsible for ROS scavenging, including *peroxidase 12* (*PRX12*), *PRX17* and *PRX60*, were downregulated in *TIP4;3*‐OE lines and upregulated in *tip4;3* mutants compared to the WT (Figure 5f). These results suggest that the various *TIP4;3* genotypes have distinct sensitivities to cold stress, which result in distinct transcriptional responses of *COR* genes, probably through an indirect mechanism.

### TIP4; 3 facilitates ROS accumulation under cold stress

**Figure 6:**
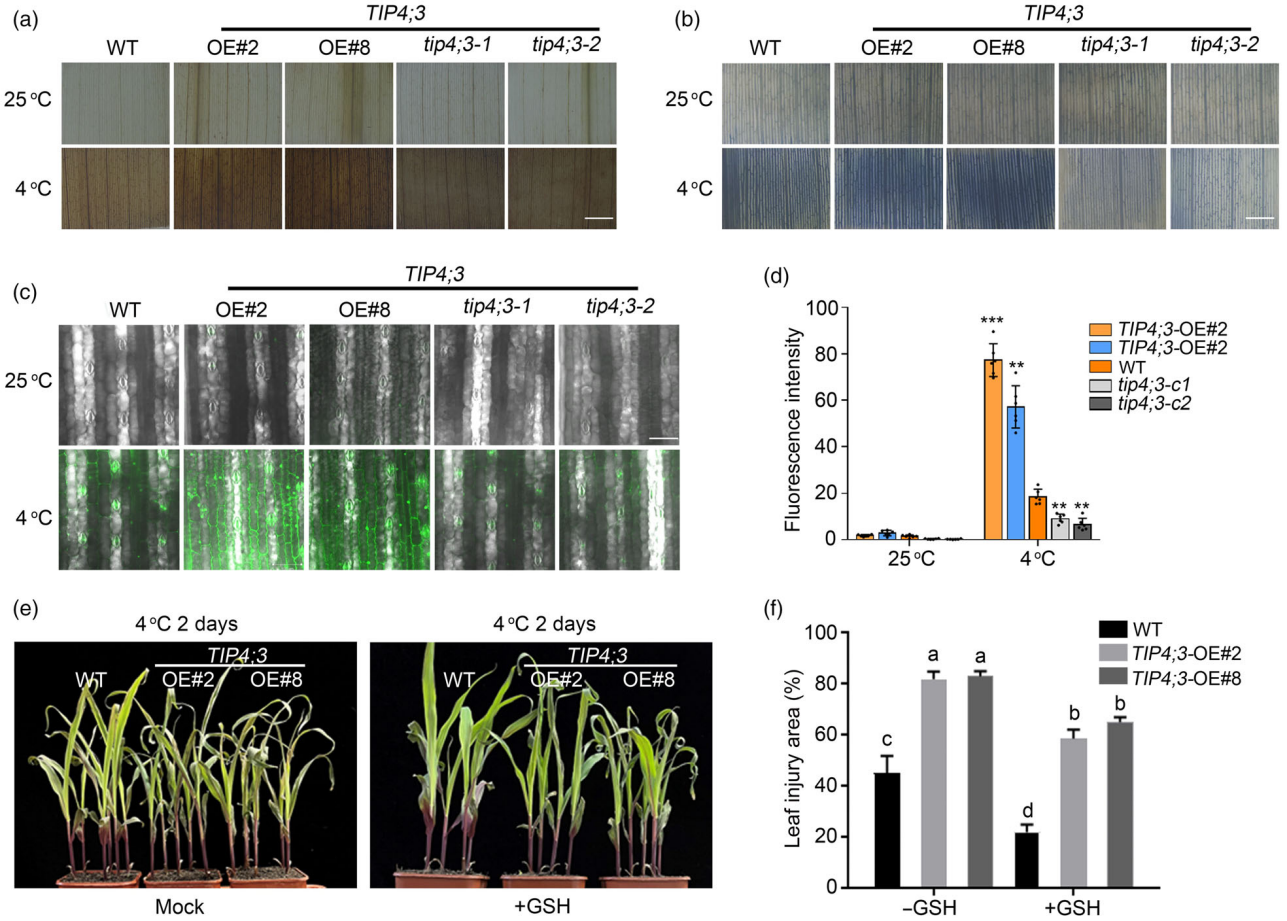
TIP4;3 affects ROS accumulation under cold stress. (a, b) Diaminobenzidine (DAB) and nitroblue tetrazolium (NBT) histochemical staining of WT, *TIP4;3*‐OE and *tip4;3* leaves under cold stress. The experiments were independently repeated three times. Representative photographs of leaf histochemical staining are shown. Scale bars, 1 mm. (c) Imaging of ROS levels in WT, *TIP4;3*‐OE and *tip4;3* leaves under 25 °C and 4 °C conditions detected with H_2_DCFDA. (d) Quantification of ROS levels in (c). Error bars represent mean ± SD (standard deviation) from three independent experiments (*n* = 10). ***P < 0.001, **P < 0.01, *P < 0.05 (two‐sided *t*‐test). (e) Exogenous application of glutathione (GSH) partially rescues the compromised cold sensitivity of *tip4;3* mutants. Maize seedlings were treated with 10 mM GSH under 4 °C for 2 days. Representative photographs were taken after 2 days of recovery. (f) Leaf injury area for the seedlings in (e). Error bars represent mean ± SD (standard deviation) from 3 biological replicates (*n* = 5 plants for each replicate). Different letters represent significant differences (*P* < 0.05) determined by one‐way ANOVA with Tukey's multiple comparisons test.

Given that TIP4;3 impacts on the expression of ROS‐scavenging genes, we speculated that TIP4;3 may influence the accumulation of ROS in response to cold stress. Indeed, overexpressing *TIP4;3* led to a substantial induction of ROS accumulation in maize, particularly under cold conditions, as evidenced by 3′3′‐diaminobenzidine (DAB) and nitroblue tetrazolium (NBT) staining (Figure 6a,b). Furthermore, cytoplasmic ROS levels were evaluated using the ROS probe 2′,7′‐dichlorodihydro‐dichlorofluorescin diacetate (H_2_DCF‐DA). We observed little difference in ROS signals among all genotypes tested in the absence of cold treatment (Figure 6c,d). However, after cold treatment, *TIP4;3*‐overexpressing plants exhibited dramatically increased cytosolic ROS accumulation, while *tip4;3* mutants showed lower cytosolic ROS accumulation compared to the WT (Figure 6c,d). Additionally, the application of glutathione (GSH), a ROS‐scavenging reagent, partially alleviated the cold injury symptoms induced by cold stress (Figure 6e,f), indicating the involvement of ROS in cold‐induced injury. These results suggest that TIP4;3 facilitates over‐accumulation of ROS under cold conditions.

## Discussion

Plants contain the largest number and maximum diversity of aquaporin homologues with diverse subcellular localization patterns, solute specificity and gating properties (Afzal *et al*., 2016). As primary water transporter proteins, TIPs are important in maintaining water balance, regulating stomatal movement, and responding to environmental stresses (Afzal *et al*., 2016). The physiological roles of AQPs throughout plant growth and development have been intensively studied during the last few decades (Alexandersson *et al*., 2005; Maurel, 2007; Maurel *et al*., 2002, 2015; Maurel and Chrispeels, 2001). However, the contribution and specific roles of TIPs in cold stress remain unclear. In this study, we provide physiological and genetic evidence showing that maize TIP4;3 compromises cold tolerance by suppressing stomatal closure and increasing water loss through transpiration, potentially leading to dehydration stress under cold conditions. The identification of genetic variation associated with AQPs may provide targets for improving cold tolerance in maize.

Previous studies have reported that the proper expression and abundance of AQPs are crucial for plant adaptation to low temperatures (Lee *et al*., 2005; Tyerman *et al*., 2002; Verdoucq *et al*., 2008). To explore the natural variation of *AQP* genes, we employed gene‐based association analysis and identify a causative variation for cold tolerance at the *TIP4;3* locus, where a 328‐bp CACTA‐like element insertion in the promoter region was found to increase histone methylation, reduced the expression of *TIP4;3*. Previous study showed that TEs are abundant in plant genomes and can be transcriptionally activated by cold stress (Chang *et al*., 2020; Liang *et al*., 2021; Makarevitch *et al*., 2015). Given that *TIP4;3* expression was notably lower in *TIP4;3* ^Hap2^ compare to *TIP4;3* ^Hap2^ under cold treatment, we propose that the CACTA‐like element may serve as a cold stress‐responsive TEs that leading to the difference in *AQP* gene expression through epigenetic regulation.

Under cold conditions, plants undergo water deficit and develop a survival strategy to prevent water loss through reduced root hydraulic conductivity, and stomatal closure (Maurel *et al*., 2008; Muraoka *et al*., 2023). In rice, the down‐regulation of *AQP* gene expression is associated with the decrease in Lpr under cold stress (Murai‐Hatano *et al*., 2008). Similarly, in cucumber and maize, the reduction in Lpr due to low temperatures might be attributed to aquaporin dysfunction (Aroca *et al*., 2005; Lee *et al*., 2004, 2005). These findings collectively establish a connection between the cold‐induced decrease in Lpr and AQP activity in various plant species. In our study, we observed a continuous decrease in the expression of several *TIP* genes, *TIP2;1*, *TIP3;2* and *TIP4;3*, in maize leaves following cold treatment (Figure 2b). Also, we found the overexpression of *TIP4;3* in plants resulted in an elevated Lpr, whereas *tip4;3* mutation showed a decreased Lpr. This suggests that the downregulation of *TIP* genes could confer a cold‐conferred decrease in Lpr, which is crucial for modulating the water permeability of the tonoplast and thereby minimizing water loss of maize seedlings exposed to cold stress.

Maintaining optimal stomatal function is essential to prevent excessive water loss while ensuring supply of carbon dioxide for photosynthesis. AQPs are likely to have impacts on crop physiological performance under stress conditions through their effects on water transport, and ultimately stomatal conductance (Moshelion *et al*., 2015). In this study, we noticed that stomatal conductance and transpiration rate were higher in *TIP4;3*‐OE plants but lower in *tip4;3* mutants compared to the WT with or without cold treatment (Figure 4h,i). Similarly, overexpression of aquaporin genes such as *TIP2;1* in grapevine, *NtAQP1* in tobacco and *HvPIP2;1* in barley increased stomatal conductance and transpiration rates (Hanba *et al*., 2004; Pou *et al*., 2013; Uehlein *et al*., 2003). All these findings support the notion that AQPs‐mediated adjustment in tissue hydraulics can have an indirect impact on stomatal conductance and transpiration (Maurel *et al*., 2016).

**Figure 7:**
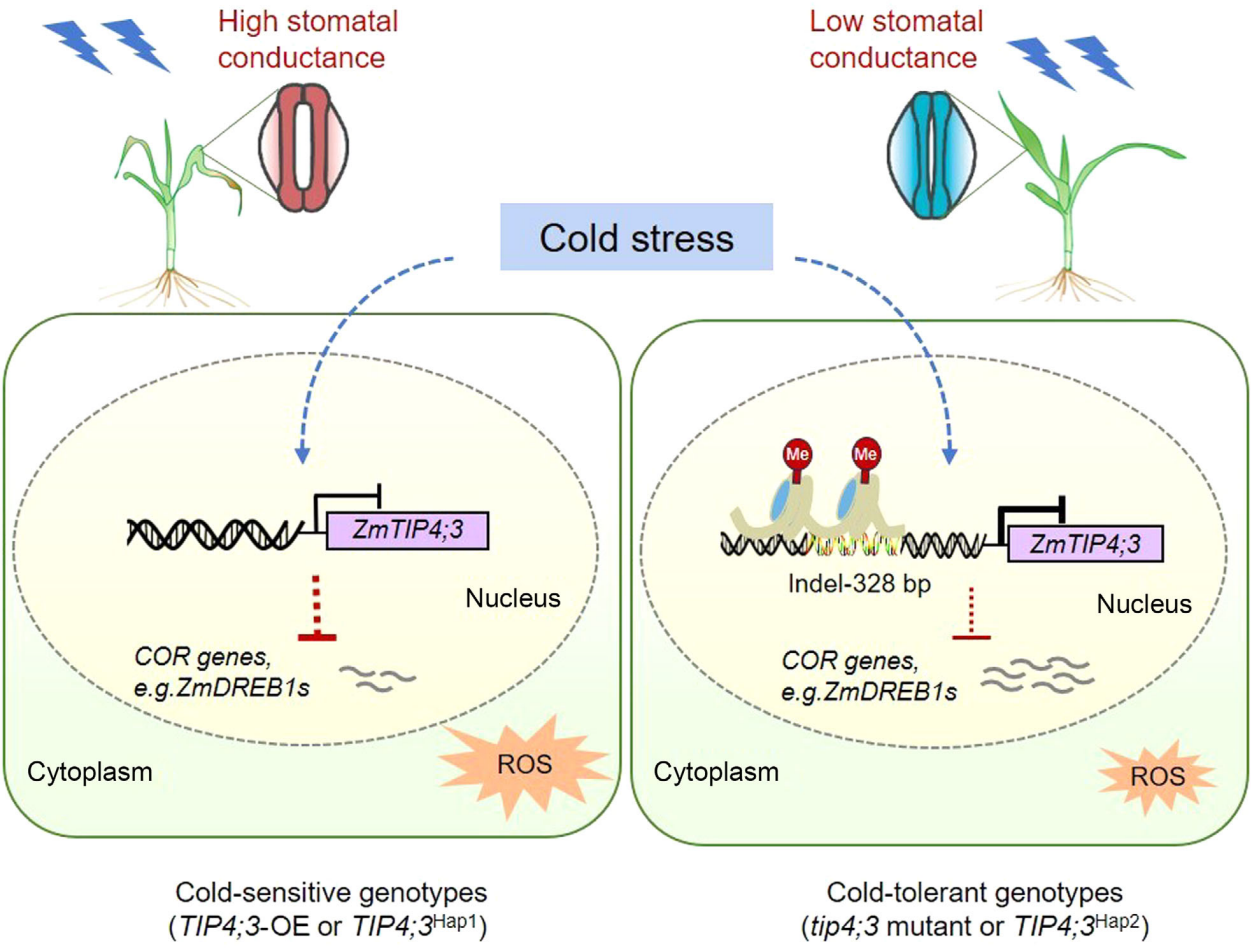
Model of TIP4;3‐mediated cold tolerance in maize. Under cold stress conditions, TIP4;3, a tonoplast aquaporin, suppresses stomatal closure and facilitates reactive oxygen species (ROS) accumulation, as well as downregulates the expression of cold‐responsive genes, thereby negatively regulating maize cold tolerance. Hap2 maize inbred lines harbour a 328‐bp transposon insertion in the *TIP4;3* ^Hap2^ promoter. This insertion increases the level of histone modifications H3K9me2 and H3K27me3, contributing to the repression of *TIP4;3* expression. Thus, *TIP4;3* ^Hap2^ inbred lines show lower *TIP4;3* expression and better cold tolerance than *TIP4;3* ^Hap1^ inbred lines.

Many studies have reported increased accumulation of ROS under cold stress. AQPs are known to be involved in the transport of H_2_O_2_, one of the best characterized ROS (Cruz de Carvalho, 2008). Generally, it is considered that TIPs are involved in the import of H_2_O_2_ into the vacuoles and improve antioxidative system to protect plants (Shivaraj *et al*., 2021). Meanwhile, ROS promotes the internalization of AQPs, thereby reducing their density at the cell surface and hydraulic conductivity (Boursiac *et al*., 2008; Wudick *et al*., 2015). This mechanism could provide feedback regulation where AQPs contribute to the initial steps of ROS signalling (Maurel *et al*., 2021). In this study, we showed that plants overexpressing *TIP4;3* had an elevated accumulation of cytosolic ROS, while *tip4;3* mutants exhibited reduced levels compared to the wild type under cold stress. Given the importance to ROS in maize hydraulic conductance and cold tolerance (Aroca *et al*., 2001), it is speculated that in cold‐sensitive genotypes with constitutive high Lpr, such as *TIP4;3*‐overexpressors and *TIP4;3* ^Hap1^, displayed an increased sensitivity to cold‐induced dehydration, leading to ROS burst and possibly membrane damage. In contrast, in cold‐tolerant genotypes, such as *tip4;3* and *TIP4;3* ^Hap2^, the reduced Lpr helps preserve water permeability and maintain ROS homeostasis, thus enhancing the cold tolerance of maize (Figure 7).

TIPs generally play roles in the water‐stress response during drought, salinity and osmotic stresses (Afzal *et al*., 2016). In this study, we observed that *tip4;3* mutants exhibited enhanced cold tolerance during the germination and seedling stages (Figure 3). Intriguingly, these mutants also showed increased drought tolerance, with higher survival rates than wild‐type plants under drought conditions (Figure S6). These enhanced stress tolerances did not compromise yield‐related traits, such as hundred‐grain weight, ear row number, or ear length, in field tests (Figure S7). This study thus provides valuable genetic resources for developing new maize varieties with both cold and drought tolerance without yield penalties.

## Methods

### Plant materials and growth conditions

The maize (*Zea mays* L.) transgenic plants were obtained from the Center for Crop Functional Genomics and Molecular Breeding, China Agricultural University. All constructs were transformed into inbred line LH244 (wild type) using Agrobacterium‐mediated transformation (Lee and Zhang, 2014). For association analysis, we used 195 maize natural variation panel, which consists of inbred lines from tropical and temperate backgrounds (Li *et al*., 2013). Maize seeds were planted in pots (30 × 20 × 15 cm) containing plant ash, vermiculite and Pindstrup soil mix (Denmark) (3:1:1) and grown at 25 °C under a 16 h light/8 h dark photoperiod with 200 μmol/m^2^/s white light and 60% relative humidity. For cold treatment, maize plants were grown for 14 days with irrigation and exposed to 4 °C under a 16‐h light/8‐h dark photoperiod for 2–4 days using a cold chamber (Conviron CMP6010). After cold treatment, the seedlings were recovered at 25 °C for 2 days prior to photography. For germination assays, dry seeds were hydrated at 25 °C for 24 h and then transferred to a glass dish containing a wet filter paper for germination in an incubator at 25 °C or 12 °C for the indicated period.

### Physiological analyses

The relative injured area was measured as described previously (Zeng *et al*., 2021). The water content was measured as follows: the seedlings were recovered at 25 °C for 2 days after cold treatment, all the above‐ground parts of the seedlings were collected and weighed to obtain the fresh weight (Wf). Subsequently, the collected samples were dried in a 70 °C oven and weighed to obtain the dry weight (Wd). The water content percentage was calculated using the formula: (Wf − Wd)/Wf × 100%.

### Plasmid construction and plant transformation

The transgenic plants (*TIP4;3‐OE#2*, *‐OE#8*) used in this study were derived from maize inbred line LH244. These constructs were generated by cloning *TIP4;3* coding sequences into *pBCXUN* (Li *et al*., 2022). To generate mutants of *tip4;3* using CRISPR‐Cas9 genome editing, fragments of the first intron were selected as guide RNA targets and cloned into *pBUE411* vector (Xing *et al*., 2014). Transgenic plants were obtained by Agrobacterium‐mediated transformation (Lee and Zhang, 2014). The promoter sequences of 1.0‐kb CIMBL52 (Hap2), 672 bp B73 (Hap1, deleting 328 bp), and 489 bp CIMBL52Δ were amplified and cloned into *pGreenII0800‐LUC* vectors using in‐fusion PCR cloning systems (C112‐02; Vazyme, Nanjing, China) to obtain the *pTIP4;3* ^B73^ *:LUC*, *pTIP4;3* ^CIMBL52^ *:LUC* and *pTIP4;3* ^CIMBL52Δ^ *:LUC*, respectively. These three plasmids were purified by Plasmid maxiprep kit (EM123‐01; Transgen, Beijing, China) and transformed separately into maize protoplast to measure the transcriptional activation assay (Li *et al*., 2022).

### RT‐qPCR and RNA‐seq

RT‐qPCR and RNA‐seq were conducted following established protocols (Zeng *et al*., 2021). Total RNAs were extracted from the leaves of 14‐day‐old maize plants using TRIzol reagent (Invitrogen, Carlsbad, CA, USA) and reverse transcribed into cDNA with M‐MLV reverse transcriptase (Promega, Shanghai, China). The expression levels target genes were detected using SYBR Green reagent (Mei5bio, MF013‐01) in a StepOnePlus Real‐Time PCR System (Applied Biosystem, Waltham, MA, USA). The maize *Ubi* gene was used as an internal control. Primer sequences for qPCR are listed in Table S5. Each experiment was independently repeated three times (three biological replicates).

For RNA‐seq assays, 14‐day‐old seedlings of wild type and *TIP4;3‐OE* grown at 25 °C were subjected to treatment before and after exposure to 4 °C for 12 h. Total RNAs were extracted from pooled wild‐type or *TIP4;3‐OE* leaves using Trizol reagent. For each sample, leaves from three individual plants were harvested. Two independent biological replicates were performed. The RNA samples were then subjected to Illumina HiSeq deep sequencing (Illumina HiSeq 6000; Illumina, San Diego, CA, USA). The generated reads were initially processed to remove adapter sequences and low‐quality reads (Q score < 20). Subsequently, the reads were mapped to the Maize genome (B73 RefGen_v4, AGPv4) using HISAT2 (v.2.2.0) with default parameters. The read counts for each gene were obtained using Feature Counts (v.2.0.1). GO enrichment analysis of differentially expressed gene (DEG) clusters was performed using the program Phyper (http://www.geneontology.org/). The significance of the GO terms was corrected using false discovery rate (FDR) < 0.05. A heatmap of the expression levels of the DEGs was constructed using TBtools software (Chen *et al*., 2020).

### Subcellular localization

Subcellular localization of TIP4;3 protein was performed as described previously (Zeng *et al*., 2021). Briefly, *pSuper:TIP4;3‐GFP* and *AtTIP1;1‐RFP* were transformed into maize protoplasts and incubated at 25 °C for 16 h. GFP and RFP fluorescence were observed by confocal microscopy (ZEISS710; Carl Zeiss, Oberkochen, Germany) with exciting light 488 and 561.

### Transient expression assays in maize protoplasts

Dual‐LUC assays was performed as described previously (Li *et al*., 2022). *pTIP4;3* ^B73^ *:LUC*, *pTIP4;3* ^CIMBL52^ *:LUC* and *pTIP4;3* ^CIMBL52Δ^ *:LUC* were used as reporter genes, REN gene driven by 35S promoter in pGreenII0800‐LUC was used as an internal control. Vectors were co‐transformed into maize protoplasts and incubated at 25 °C for 16 h. The luciferase signal was measured via the Dual‐Luciferase Reporter Assay system (Promega) on a GLOMAX 20/20 luminometer (Promega, Madison, WI, USA). Relative LUC activity was calculated by normalizing LUC activity to REN activity.

### Histochemical detection of H_2_O_2_ , O^2^ ^−^· and ROS

Two‐week‐old maize seedlings were grown in mixed soil in a greenhouse and then treated with 4 °C for 12 h. H_2_O_2_ and O^2−^· was stained with 3′3′‐diaminobenzidine (DAB, Sigma‐Aldrich, St. Louis, MO, USA) and nitroblue tetrazolium (NBT, Beyotime, Shanghai, China), respectively, as described previously (Jiang *et al*., 2022). For DAB staining, leaf pieces were incubated in 1 mg/ml DAB for 10 h, decolorized with 80% alcohol until the decolorizing solution was colourless and then observed under an Olympus microscope (SZX16). For NBT staining, leaf pieces were immersed in 10 mL NBT staining solution (6 mM diluted with potassium phosphate) for 10 h, decolorized with 80% ethanol and then observed under an Olympus microscope (SZX16). The ROS level in guard cells was detected using H_2_DCFDA staining as described (Gao *et al*., 2022). Portions of leaves were immersed in staining buffer (10 mM Tris–HCl, 50 mM KCl, 50 μM H_2_DCFDA, 0.02% Tween‐20 at pH 7.2) and subjected to vacuum filtration for 20 min at room temperature in the dark. The leaves were then washed with distilled water to remove excess dye. Fluorescence was examined using a confocal laser‐scanning microscope (ZEISS710).

### TIP4;3‐based association analysis

According to the reference sequences of B73, the genomic regions of *TIP4;3* from 195 maize genotypes were amplified and sequenced (Yang *et al*., 2010). The sequences were aligned by MEGA 7.0, and DNA variations among inbred lines were identified. 195 maize inbred lines were analysed for the association between the genetic variations in *TIP4;3* and the relative injured area. The standard mixed linear model was applied (Yu *et al*., 2006), in which the population structure (Q) and kinship (K) were estimated for *TIP4;3*‐based association analysis. *P* value was calculated by the mixed linear model (MLM). SNP loci association with the phenotype and pairwise linkage disequilibrium was calculated by TASSEL software (Bradbury *et al*., 2007; Zhang *et al*., 2010).

### Stomatal aperture analysis

The stomatal aperture assay was conducted as described (Guo *et al*., 2023). In brief, a 1‐cm piece of the first fully expanded leaf from 8‐day‐old maize seedlings was excised in the middle of leaf and immersed in stomatal opening solution MES buffer (10 mM MES–KOH, pH 5.7, 10 mM KCl, and 50 μM CaCl_2_) in the light (150 μmol/m^2^/s^2^) for 3 h at 25 °C to open the stomata. Then, 12 μM ABA was added into the MES buffer for another 2 h. The abaxial epidermis of leaf was evenly coated with colourless nail polish. After drying, the nail polish was peeled off with transparent scotch tape. Stomata sticking to the tape were photographed using an Olympus BX53 microscope (Olympus, Tokyo, Japan). The stomata were observed with a 40‐fold objective lens. Stomatal apertures were measured using the ImageJ software. Approximately 200 stomatal apertures from each seedling were measured in each experiment.

### Osmotic water permeability assay

The osmotic water permeability assay was conducted as described (Zhang and Verkman, 1991). Oocytes were transferred 2–3 days after mRNA injection from Barth's solution (200 mosM/kg) at room temperature to the same solution diluted to 40 mosM/kg with distilled water. Changes in cell volume were monitored using a microscope (Nikon Ti‐U), and photographs were taken at 30‐ to 45‐s intervals. Oocyte diameters were measured four times along two sets of perpendicular axes. The volume V was estimated as the mean of two ellipsoid volumes. The osmotic permeability coefficient (Pf) was calculated using the formula: Pf = Vo[d(*V*/*V*_O_)/d*t*]/[*S* × Vw (Osm_in_‐Osm_out_)], with initial oocyte volume *V*_O_ = 9 × 10^−4^ cm^3^, initial oocyte surface area *S* = 0.045 cm^2^ and molar volume of water Vw = 18 cm^3^/mol, respectively.

### Root hydraulic conductivity (Lpr) assay

Root hydraulic conductivity (Lpr) was evaluated using the pressure chamber method, which was calculated by the slope of the root water flow rate curve at different pressures as described (He *et al*., 2023). Four‐week‐old maize plants grown hydroponically were used for the assay. The first leaf base was cut from maize plants under hydroponic conditions. The entire root system was placed in a sealed pressure chamber, with the incision exposed outdoors through a gasketed hole. Pressure was gradually increased from 0.1 to 1.2 MPa, with measurements taken at intervals of 0.1 MPa. A 1.5‐mL centrifuge tube (EP tube) filled with absorbent paper was used to collect the effluent juice at least three times at each pressure for 1 min each time. The mass of EP tube was weighed before and after water absorption, and the xylem fluid flow per unit time (Q, mg/min) was calculated when the stable flow rate was reached under each pressure. Roots were then cleaned, dried, and weighed. The amount of effluent juice collected per unit root weight and per unit outflow time (water flow rate, Jv, mg/(g·min)) was calculated. The slope of the curve relating water flow rate to pressure represents the root water conductivity (Lpr).

### GSH treatment assays for cold tolerance

Fourteen‐day‐old seedlings were grown in pots containing equal‐weight soil for the GSH treatment assays as described (Jiang *et al*., 2022). Young maize seedlings were deprived of water for 3 days before the treatment. The equal amounts of 10 mM GSH solution were applied to soil‐grown plants. After the 12‐h treatment, plants were exposed to cold treatment at 4 °C for 2 days. At least five individual plants were compared with distilled water‐treated (mock) plants for each test. The statistical data based on obtained data from three independent experiments were collected.

### Stomatal conductance and transpiration rate assays

The third leaf of the 21‐day‐old seedlings of wild type (WT), *TIP4;3‐OE* and *tip4;3* mutant was used to measure stomatal conductance and transpiration rate by LI‐6400XT portable photosynthesis measurement system as described (He *et al*., 2023). The greenhouse humidity was between 60% and 65%; CO_2_ concentration was stabilized using CO_2_ cylinders. Four plants were tested for each genotype and three biological replicates were carried out.

### Construction of phylogenetic tree

Amino acid sequences of *TIP4;3* homologues were downloaded from Gramene protein database (https://www.gramene.org/). MEGA7.0 was used to build the phylogenetic tree. The numbers at each branch of the tree mean the bootstrap value.

### Data significant test

Significant differences were analysed by Student's *t*‐test or Tukey's test.

## Conflict of interest

The authors declare that the research was conducted in the absence of any commercial or financial relationships that could be construed as a potential conflict of interest.

## Author contributions

Y.S., conceived, designed, and directed the project. R.Z., and X.Z., performed most of the experiments. S.Z., performed the maize transformation. G.S., and Q.L., performed yield measurement. R.Z., M.L., Z.Z., and D.F., performed association and RNA‐seq analyses. R.Z., X.Z., L.G., F.T., S.Y., and S.Y., analysed the data. R.Z., S.Y., and Y.S., wrote the manuscript with comments from all authors.

## Supporting information


**Figure S1** Expression of *TIP* family genes in maize response to cold stress.
**Figure S2** Schematic diagram of the 328 bp sequence.
**Figure S3** Identification of NIL lines of *TIP4;3* and *TIP4;3* mutants.
**Figure S4** Phenotypic testing of two alleles of *tip4;3* mutants.
**Figure S5** Representative photographs of oocytes that were injected with the cRNA of *TIP4;3*, *AtTIP1;1* (a positive control), H_2_O (a negative control) after switching from isotonic to hypotonic buffer.
**Figure S6** *TIP4;3* negatively regulates drought tolerance in maize.
**Figure S7** Yield‐related traits of *tip4;3* mutant lines.

**Table S1** Leaf injury area and haplotype of 195 maize inbred lines.

**Table S2** A total of 2,620 differentially expressed genes (DEGs) based on the criteria of a significant difference (*P* < 0.05) with an absolute fold‐change ≥ 2 identified in WT seedlings after exposure to cold tratment.

**Table S3** 255 upregulated genes and 737 downregulated genes were identified under permissive conditions (25°C) in *TIP4;3*‐OE lines compared to the wild type.

**Table S4** 759 TIP4;3‐induced genes and 441 TIP4;3‐repressed genes were identified under cold treatment in *TIP4;3*‐OE compared to the wild type.

**Table S5** Primers used in this study.

## Data Availability

The raw RNA‐seq data in this paper have been deposited at NCBI under Bioproject with the accession number PRJNA1054219.
