# Supplementary material for: Genetic variation in the aquaporin TONOPLAST INTRINSIC PROTEIN 4;3 modulates maize cold tolerance
Source: Plant Biotechnol J. 2024 Jul 18;22(11):3037–50. doi: 10.1111/pbi.14426 (PMC11500999; doi:10.1111/pbi.14426)
Supplement: Supplementary file 1 — Figure S1 Expression of TIP family genes in maize response to cold stress. Figure S2 Schematic diagram of the 328 bp sequence. Figure S3 Identification of NIL lines of TIP4;3 and TIP4;3 mutants. Figure S4 Phenotypic testing of two alleles of tip4;3 mutants. Figure S5 Representative photographs of oocytes that were injected with the cRNA of TIP4;3, AtTIP1;1 (a positive control), H2O (a negative control) after switching from isotonic to hypotonic buffer. Figure S6 TIP4;3 negatively regulates drought tolerance in maize. Figure S7 Yield‐related traits of tip4;3 mutant lines. [file PBI-22-3037-s003.docx]

**Supplemental data**


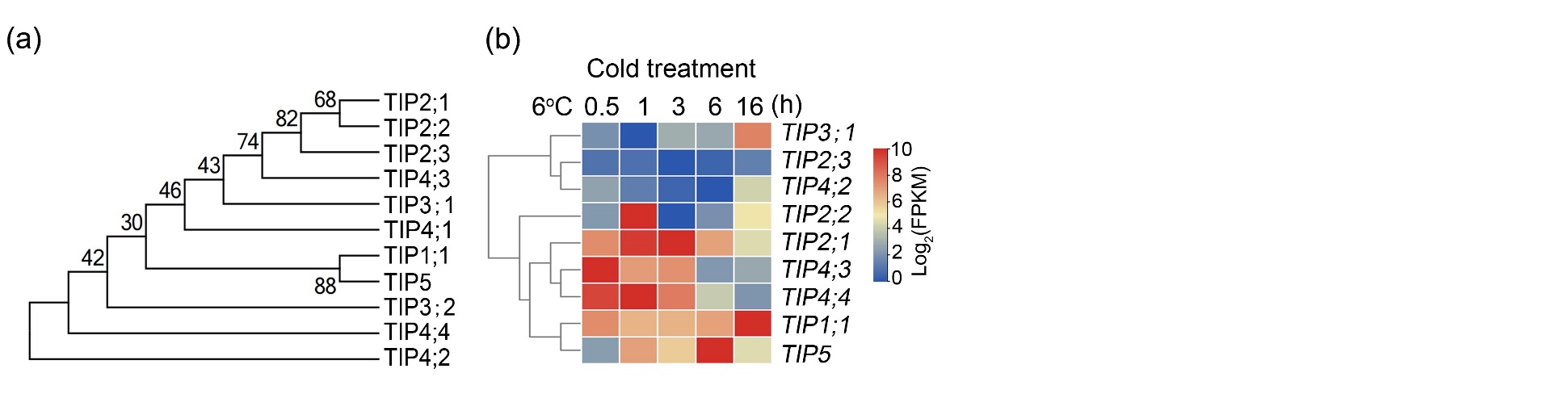


Figure S1 Expression of TIP family genes in maize response to cold stress.

(a) The phylogenetic relationship among TIP family members in maize is depicted in the tree.

(b) Heatmap displays the expression levels of 9 out of 11 maize TIP family genes in response to cold stress.


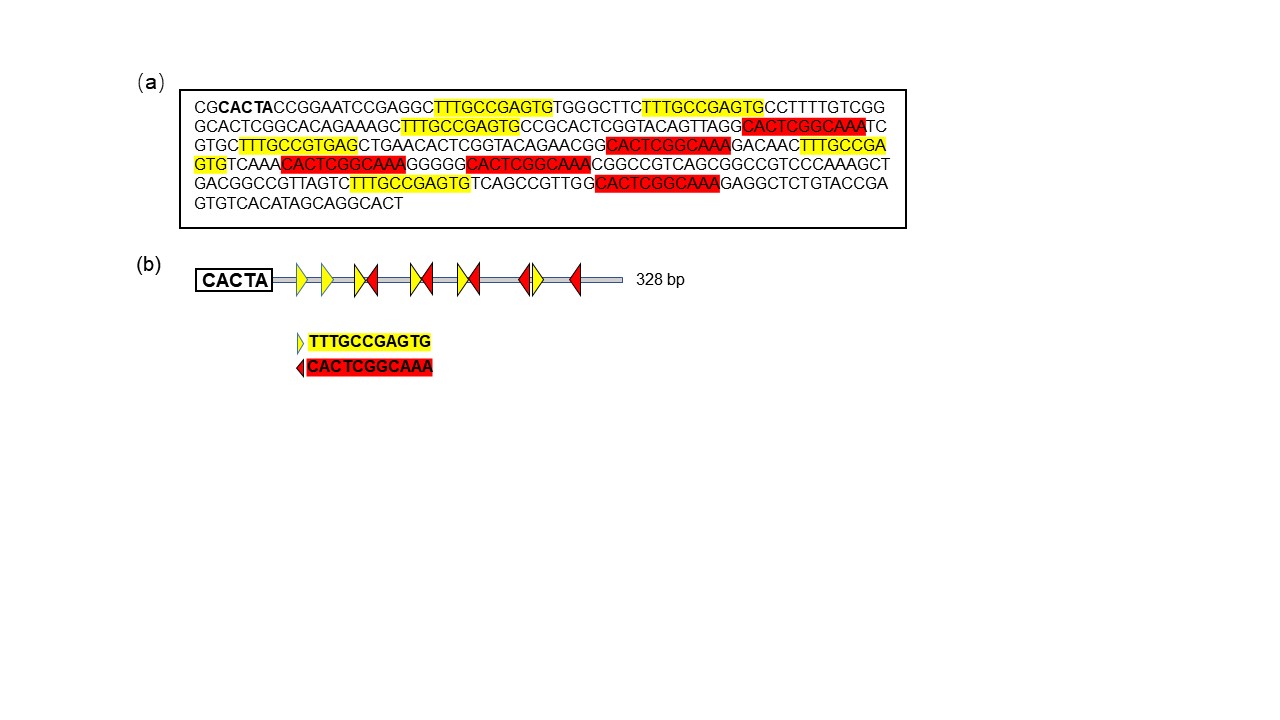


Figure S2 Schematic diagram of the 328 bp sequence

(a-b) The nucleotide sequences of the 328-bp (a) and the structure of the CACTA-like transposon (b). Terminal inverted repeat (TIR) sequences are also shown. Yellow and red arrows indicate forward and reverse duplications of sub-terminal repeats (STRs), respectively.


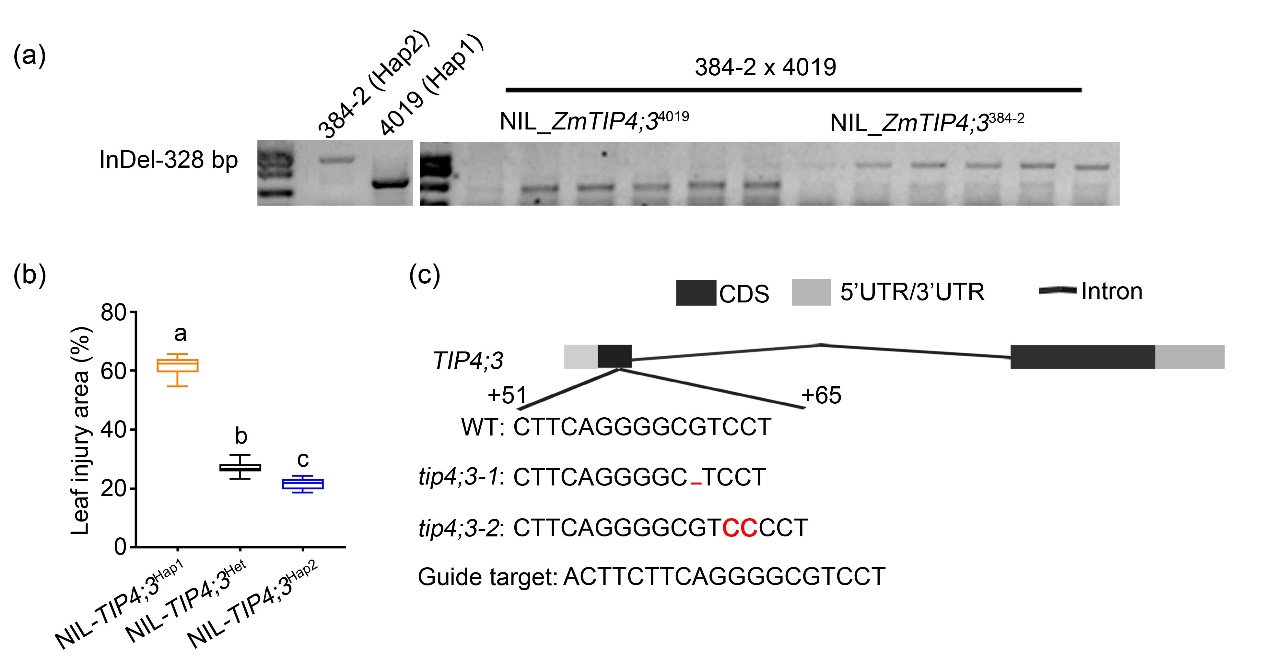


Figure S3 Identification of NIL lines of *TIP4;3* and *TIP4;3* mutants.

(a) The selection of segregating NILs carrying a homozygous allele of either *TIP4;3*^384-2^ (Hap2) or *TIP4;3*^4019^ (Hap1)using the molecular marker (Indel-328).

(b) Leaf injury area analysis of homozygous *TIP4;3*^Hap1^, *TIP4;3*^Hap2^ and heterozygous *TIP4;3* after cold treatment. Different letters represent significant differences (*P* < 0.05, one-way ANOVA).

(c) CRISPR/Cas9 technology was used to generate two mutant alleles, *tip4;3-1* and *tip4;3-2.* The gene structure of *TIP4;3* and the sequences of guide target are shown.

**
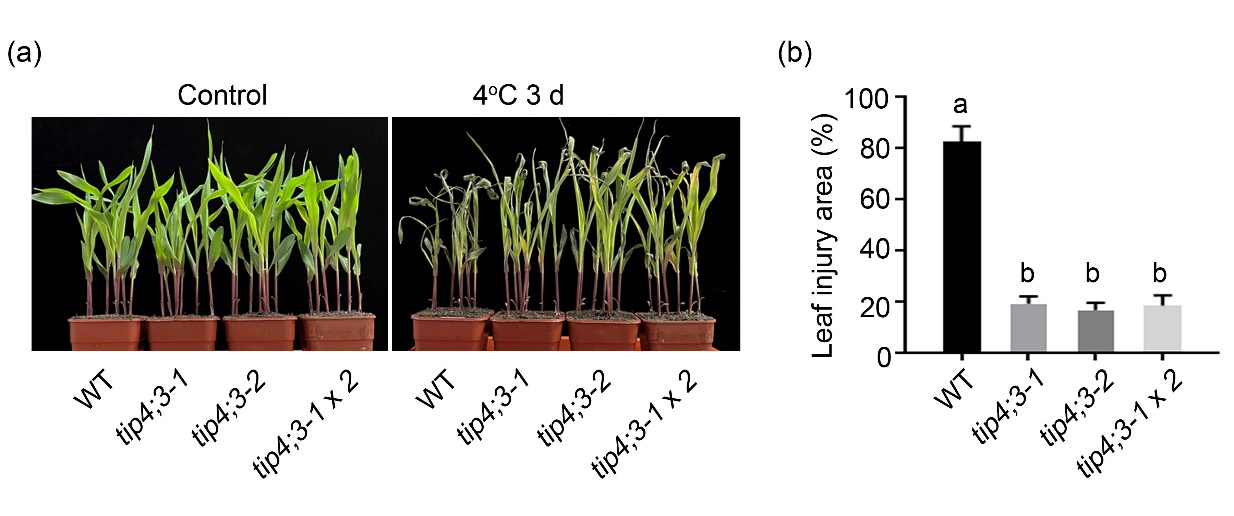
**

Figure S4 Phenotypic testing of two alleles of *tip4;3* mutants.

(a-b) Cold tolerance phenotype (a) and leaf injury area (b) of wild-type, *tip4;3-1*, *tip4;3-2* and *tip4;3-1* × *tip4;3-2* (F1 plants produced by crossing *tip4;3-1* with *tip4;3-2*) under cold conditions. Fourteen-day-old seedlings grown at 25 °C were incubated at 4 °C for 3 d. Representative images were taken after 2 days of recovery. In (b), each bar represents the mean ± SD (standard deviation). Different letters represent significant difference at *P* < 0.05 (one-way ANOVA).


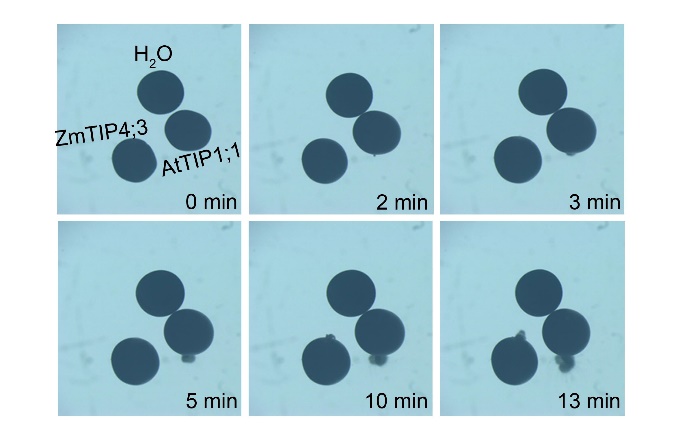


Figure S5 Representative photographs of oocytes that were injected with the cRNA of TIP4;3, AtTIP1;1 (a positive control), H_2_O (a negative control) after switching from isotonic to hypotonic buffer.

**
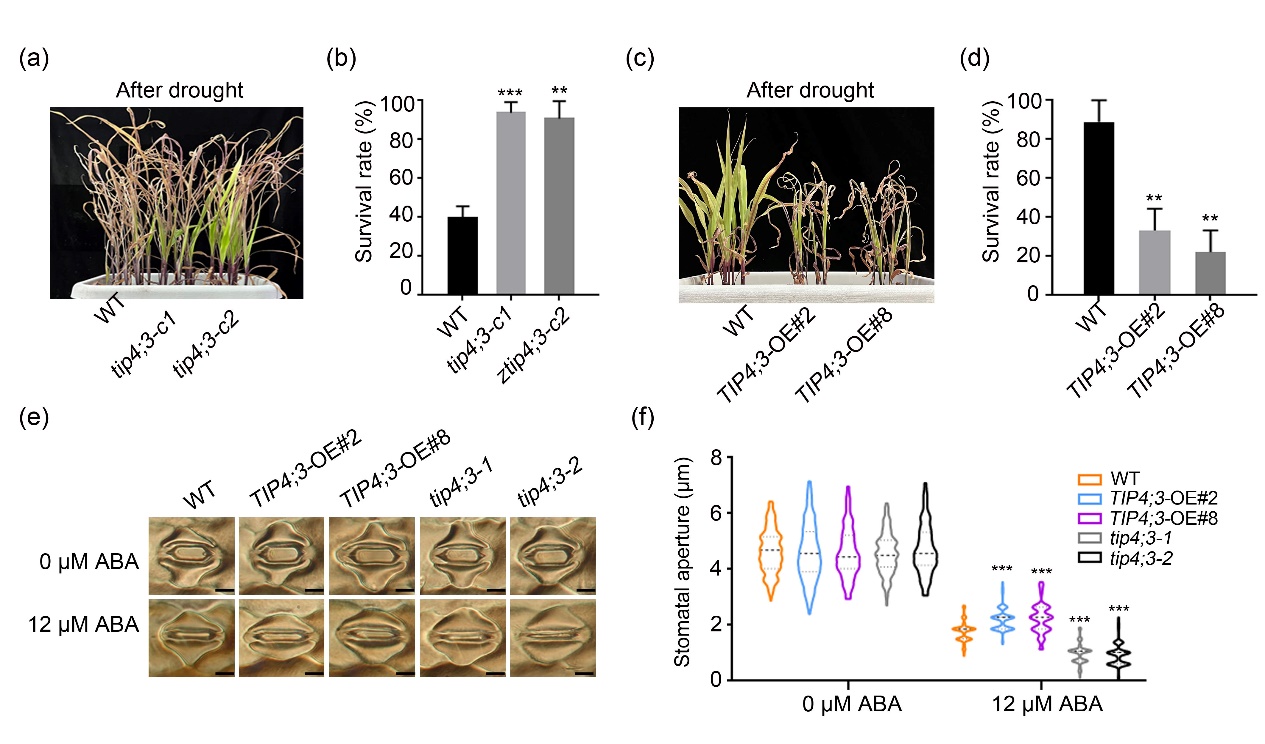
**Figure S6 *TIP4;3* negatively regulates drought tolerance in maize.

(a) Drought tolerance phenotype of *TIP4;3* mutant plants. The photograph shows the phenotype of TIP4;3 mutant plants after subjected to a drought stress for 14 days.

(b) Survival rate of WT and *TIP4;3* mutant plants after subjected to a drought stress. Each bar represents the mean ± SD from three independent biological experiments. The statistical significance was determined by a two-sided *t*-test. ** *P* < 0.01, ***, *P* < 0.001.

(c) Drought tolerance phenotype of *TIP4;3*-OE plants. Photographs were taken after the drought treatment.

(d) Survival rate of WT and *TIP4;3*-OE plants after subjected to a drought stress. Each bar represents the mean ± SD from three independent biological experiments. The statistical significance was determined by a two-sided *t*-test. ** *P* < 0.01.

(e) Representative images of stomata before and after ABA-induced stomatal closure: Images showing stomata from different genotypes (WT, *TIP4;3-*OE#2, *TIP4;3*-OE#8, *tip4;3-1*) before and after treatment with the hormone abscisic acid (ABA). The first fully expanded leaves of 8-day-old seedlings were immersed in MES-KOH buffer in light for 3 h to facilitate the opening of the stomata. Subsequently, ABA was added for another 1.5 h before being photographed. Bar, 5 μm.

(f) Quantification of stomatal aperture with or without ABA treatment. The dashed line represents the median, and the upper and lower dotted lines represent the third quartile and first quartile, respectively. The bounds of the plot represent data density. Each bar represents the mean ± SD (standard deviation). Significant differences are indicated by two-sided *t*-test. ***, *P* < 0.001.


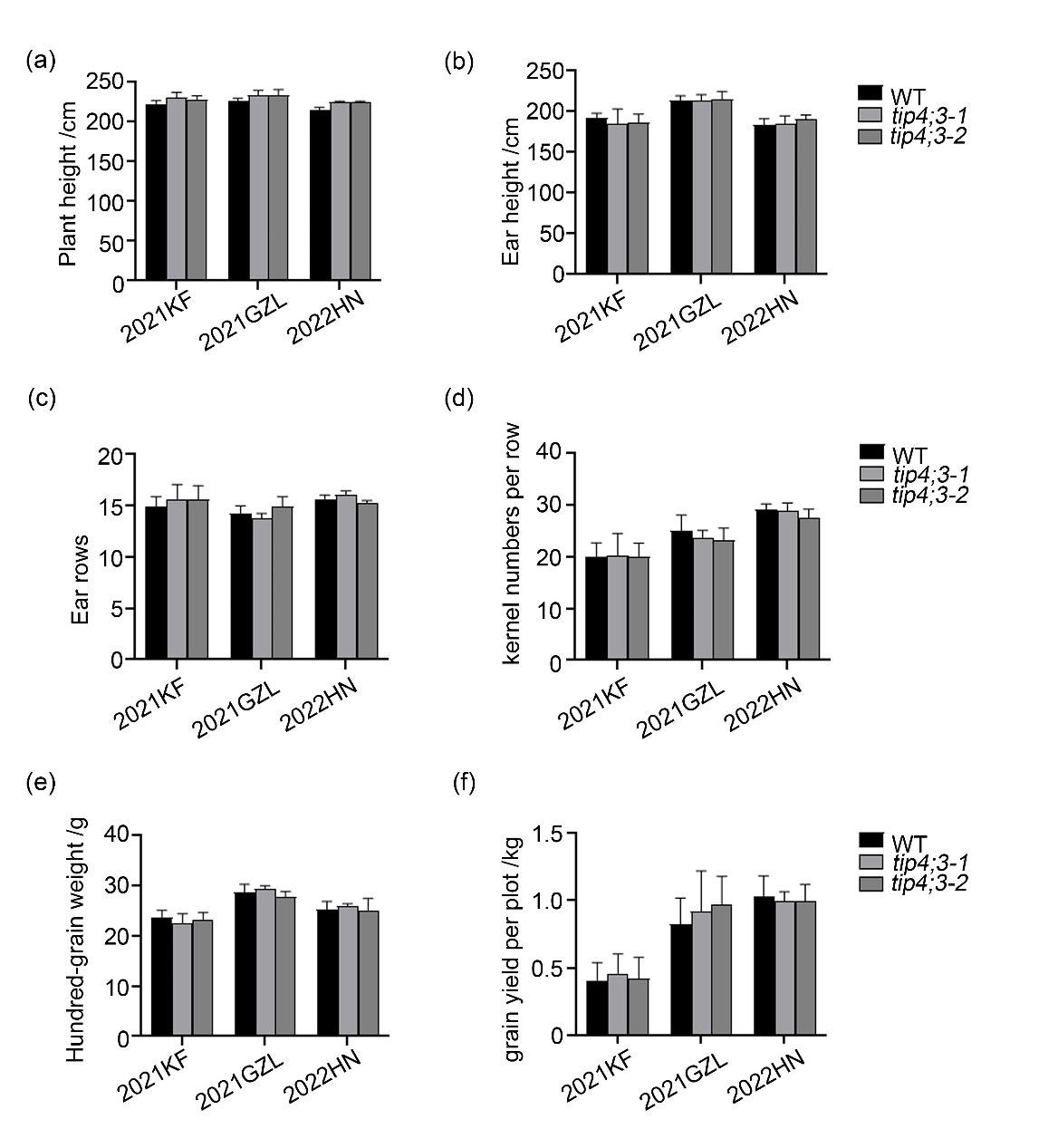


Figure S7 Yield-related traits of *tip4;3* mutant lines.

(a-f) Comparison of relative (a) plant height (b) ear height, (c) ear rows (d) kernel numbers per row (e) hundred-grain weight and (F) gain yield per plot between wild-type and *tip4;3* mutants under optimal feeding conditions. Maize was planted in Kai Feng,2021, Gong Zhuling, 2021 and San Ya, China 2022.

In a-f, bars represent the mean ± SD (standard deviation) obtained from at least 60 plants of each genotype from six independent plots. The statistical significance was determined by a two-sided *t*-test.
